# Reproducibility dataset for a large experimental survey on word embeddings and ontology-based methods for word similarity

**DOI:** 10.1016/j.dib.2019.104432

**Published:** 2019-08-26

**Authors:** Juan J. Lastra-Díaz, Josu Goikoetxea, Mohamed Ali Hadj Taieb, Ana García-Serrano, Mohamed Ben Aouicha, Eneko Agirre

**Affiliations:** a NLP & IR Research Group, ETSI de Informática (UNED), Universidad Nacional de Educación a Distancia, Juan Del Rosal 16, 28040, Madrid, Spain; b IXA NLP Group, Faculty of Informatics, UPV/EHU∖∖ Manuel Lardizabal 1, 20018, Donostia, Basque Country, Spain; c Faculty of Sciences of Sfax, Tunisia

**Keywords:** Ontology-based semantic similarity measures, Word embedding models, Information content models, WordNet, Experimental survey, HESML, Reprozip

## Abstract

This data article introduces a reproducibility dataset with the aim of allowing the exact replication of all experiments, results and data tables introduced in our companion paper (Lastra-Díaz et al., 2019), which introduces the largest experimental survey on ontology-based semantic similarity methods and Word Embeddings (WE) for word similarity reported in the literature. The implementation of all our experiments, as well as the gathering of all raw data derived from them, was based on the software implementation and evaluation of all methods in HESML library (Lastra-Díaz et al., 2017), and their subsequent recording with Reprozip (Chirigati et al., 2016). Raw data is made up by a collection of data files gathering the raw word-similarity values returned by each method for each word pair evaluated in any benchmark. Raw data files were processed by running a R-language script with the aim of computing all evaluation metrics reported in (Lastra-Díaz et al., 2019), such as Pearson and Spearman correlation, harmonic score and statistical significance p-values, as well as to generate automatically all data tables shown in our companion paper. Our dataset provides all input data files, resources and complementary software tools to reproduce from scratch all our experimental data, statistical analysis and reported data. Finally, our reproducibility dataset provides a self-contained experimentation platform which allows to run new word similarity benchmarks by setting up new experiments including other unconsidered methods or word similarity benchmarks.

**Table:** Specifications Table

Subject area	*Computer science*
More specific subject area	*Artificial Intelligence*
Type of data	Tables *in text-based CSV file format, two self-contained reproducible experiments in HESML XML-based file format and Reprozip binary file format respectively, pre-trained word embeddings in text-based vector files, and a R-language script file for data processing. Next, we detail the seven different types of data provided by this dataset: (1) word similarity datasets (benchmarks) in text-based CSV file format; (2) pre-trained word embedding files in three different text-based vector file formats; (3) HESML experimentation setup file in XML-based file format; (4) raw output similarity values returned by all methods evaluated in our companion paper which are distributed in text-based CSV file format; (5) processed output data files which contain all data tables as shown in our companion paper which are distributed in text-based CSV file format; (6) self-contained reproducible experiment file in Reprozip binary file format; and finally, (7) a R-script post-processing file to generate automatically all final data tables from raw output similarity files which are reported in our companion paper**[1]*.
How data was acquired	*Data was acquired in seven different ways as follows: (1) word similarity datasets were gathered from their primary repositories or manually transcribed from their publications, then they were normalized to lowercase and converted into text-based CSV file format; (2) pre-trained word embedding files were gathered from their primary repositories; (3) HESML experimentation setup file was manually created in XML spy; (4) raw output similarity values were created by running our enclosed HESML experimentation setup file detailing all experiments reported in our companion paper; (5) processed output data files were created by running the enclosed R-language script file on our output data files; (6) self-contained Reprozip reproducible experiment file was created by recording with Reprozip program the running of the HESML-based experiments onto a Linux-based platform detailed in appendix B of our companion paper**[1]**; and finally, (7) post-processing R-language script file was developed and validated into RStudio and R statistical programs.*
Data format	*Raw input data files made up by the collection of word similarity datasets and pre-trained word embedding files. Raw output data files containing all raw similarity values returned by our experiments. Two different reproducible experimentation files to replicate all our experiments and results in two different ways as detailed in appendix B of our companion paper**[1]**. A post-processing script whose aim is to carry-out the data analysis of the raw output data generated by our experiments and automatically generating all data tables reported in our companion paper.*
Experimental factors	*Main criteria for the creation of this data collection has been to provide a self-contained reproducibility and experimentation package including all resources needed to reproduce all our experiments on word similarity and relatedness as well as generating all raw data and final results reported in our companion paper**[1]**from the primary input data used in our experiments. A second criteria of our data collection is to provide all raw and processed data generated in our experiments**[1]**for their direct use or as a means of verification for any independent replication of our experiments or further data analysis. A third criteria is to set a self-contained experimentation platform which can be used to generate new experiments on word similarity and relatedness by evaluating unconsidered methods or benchmarks. Finally, we point out that none preprocessing is needed to use our data*.
Experimental features	*All experiments were carried-out by running a reproducible experiment file with HESMLclient program and HESML V1R4 library**[13]*.
Data source location	*e-CienciaDatos, Repositorio de datos UNED. Consorcio Madroño, Madrid (Spain)*.
Data accessibility	https://doi.org/10.21950/AQ1CVX
Related research article	Lastra-Díaz, J. J., Goikoetxea, J., Hadj Taieb, M. A., García-Serrano, A., Ben Aouicha, M., Agirre, E., (2019). A reproducible survey on word embeddings and ontology-based methods for word similarity: linear combinations outperform the state of the art. Engineering Applications of Artificial Intelligence 85, 645–665 [1].

**Table:** 

**Value of the data** • This data is useful for the research community for certain reasons as follows. First, this data significantly simplifies the development of large benchmarks on word similarity and relatedness based on ontology-based methods and word embeddings, as well as the implementation of new methods, by gathering most word similarity and relatedness benchmarks, as well as most recent and best performing ontology-based semantic similarity measures based on WordNet and pre-trained word embedding models, together with all complementary software tools (see appendix B [1]) and report generation script (post-processing R-script) into a same repository [15] and common software platform [13]. Thus, this data avoids the tedious and sometimes complex task of gathering all these aforementioned experimentation resources, as well as the integration and set up of multiple independent software libraries and tools, or a software implementation from scratch of many methods reported in the literature. Second, this data provides for the first time a fully reproducible experimental survey of ontology-based semantic similarity measures and word embeddings implemented into a common software platform, which allows an easy replication of all methods, experiments and results on word similarity and relatedness reported in our companion paper [1]. And third, this data is expected to become into a standard benchmark for this line of research as well as a development platform for new methods and experiments. • The research community in the fields of Natural Language processing (NLP), Information Retrieval (IR) and Artificial Intelligence (AI) can benefit from this data by using it in some research tasks as follows: (1) evaluation of methods for the estimation of the degree of similarity and relatedness between words; (2) evaluation and development of applications based on word similarity and relatedness methods; (3) replication of benchmarks on word similarity and relatedness, such as those introduced in our companion paper [1]; (4) development of new methods for the estimation of word similarity and relatedness; (5) further data analysis and insights by analyzing the raw similarity and relatedness values returned by all methods evaluated in our companion paper [1]; and finally, (6) teaching and training on ontology-based semantic measures and word embeddings. • This data can be used for further insights and development of experiments by editing and running our main HESML-based experimentation file (see Table 1) to set up other unexplored word similarity benchmarks and pre-trained word embedding models, as well other new or existing word similarity methods implemented in HESML software library. • Another value of this data is that it provides two self-contained and reproducible experiments based on HESML and Reprozip respectively, which are easily portable and reproducible in any Java-complaint platform, and whose reproducibility is warranted in the long-term. On one hand, HESML is a self-contained Java software library, and thus it inherits all portability and reproducibility advantages provided by the Java platform. And on the other hand, our Reprozip-based reproducible experiment file provides a further reproducibility warranty in the long-term by capturing and packaging into a same execution unit all experimentation program dependencies, being able to reproduce the packaged experiments onto any other platform regardless of the hardware and software configuration used in their creation. • Finally, a further significant value of this data is that it provides for the first time the raw similarity values returned by most of ontology-based semantic similarity methods and word embedding models proposed during the last 30 years of research up to now in the evaluation of the largest set of word similarity and relatedness benchmarks reported in the literature [1]. This data provides at least three new research possibilities to the research community as follows: (1) to carry-out further data analysis on these methods with the aim of drawing new insights; (2) the exploration of aggregated methods based on linear or non-linear combinations as preliminary explored in our companion paper [1]; and (3) the capability of validating other software implementations of the family of methods evaluated herein by comparing the raw similarity values provided herein with the values returned by the methods being validated or reproduced.

## 1 Data

Table 1 details the data files included in the reproducibility dataset [15] for word similarity and relatedness benchmarks introduced by this article. Likewise, Table 2 details all pre-trained word embedding models packaged into the “*WordEmbeddings.zip”* file, whilst Table 3 details all word similarity datasets packaged into the “*Word_Similarity_Datasets.zip”* file.

**Table 1:** Content of our reproducibility dataset which is publicly available at the UNED Dataverse repository [15].

Data filename	Description
appendix-reproducible-experiments.pdf	Copy of the appendix B of our companion paper [1] introducing a detailed protocol to use this dataset.
benchmark_survey.exp	HESML reproducible experiment file which allows to reproduce all our experiments and results by running HESMLclient.
embeddings_vs_ontomeasures_final_tables.R	A post-processing R script file which processes all raw similarity files and generates a collection of Comma Separated (CSV) files containing all data tables in our main companion paper [1].
processed_output_benchmarks.zip	This ZIP file contains all processed CSV files generated by our post-processing R script.
raw_output_benchmark_all_datasets.zip	This ZIP file contains all raw output similarity files produced by running HESMLclient program with our ‘benchmark_survey.exp’ reproducible experiment file as input. Thus, it contains all our raw experimental data.
WN_ontology_measures_vs_embeddings.rpz	Reprozip file to reproduce all our experiments in the long-term on any Reprozip compliant platform regardless the availability of the original platform used in our experiments.
WordEmbeddings.zip	This ZIP file contains all pre-trained word embedding models evaluated in our experiments.
Word_Similarity_Datasets.zip	This ZIP file contains all word similarity datasets (benchmarks) evaluated in our experiments.

**Table 2:** Pre-trained word embedding models packaged into the *WordEmbeddings.zip* file [15].

Filename	WE model	Primary source
attract-reppel.emb	Attract-repel [20]	https://github.com/nmrksic/attract-repel
fastext.emb	FastText [3]	https://github.com/facebookresearch/fastText/blob/master/pretrained-vectors.md
glove.emb	GloVe [22]	https://nlp.stanford.edu/projects/glove/
cbow.emb	CBOW [17]	https://code.google.com/archive/p/word2vec/
sp.500d.emb	SymPatterns (SP-500d) [26]	https://homes.cs.washington.edu/∼roysch/papers/sp_embeddings/sp_embeddings.html
paragram-ws.emb	Paragram-ws [28]	https://www.cs.cmu.edu/∼jwieting/
paragram-sl.emb	Paragram-sl [28]	https://www.cs.cmu.edu/∼jwieting/
cf.emb	Counter-fitting [21]	https://github.com/nmrksic/counter-fitting
wordnet-randomwalks.emb	WN-RandomWalks [9]	http://ixa2.si.ehu.es/ukb/
wordnet-ukb.ppv	WN-UKB [2]	http://ixa2.si.ehu.es/ukb/
nasari/en_wordsenses_BN.txt nasari/nasari-unified	Nasari [5]	http://lcl.uniroma1.it/nasari/

**Table 3:** Detail of the main features of all word similarity and relatedness datasets evaluated in our companion paper [1] and packaged into the Word_Similarity_Datasets.zip file. We use the following abbreviations and acronyms in table above: WordNet (WN), Similarity (Sim), Relatedness (Rel), Nouns (N), Verbs (V) and Adjectives (A).

Dataset	Content	Type	#word pairs	Filename (*.csv)
MC28 [19]	Nouns	Similarity	28	Miller_Charles_28_dataset
RG65 [25]	Nouns	Similarity	65	Rubenstein_Goodenough_dataset
PS_full_[23]	Nouns	Similarity	65	PirroSeco_full_dataset
Agirre201 [1]	Nouns	Similarity	201	Agirre201_lowercase_dataset
SimLex665 [11]	Nouns	Similarity	665	SimLex665_dataset
MTurk771 [10]	Nouns	Relatedness	771	Halawi_MTURK771_dataset
MTurk287/235 [24]	Nouns	Relatedness	235	Radinsky_MTurk287_filtered235_dataset
WS353Rel [7]	Nouns	Relatedness	245	WordSim353Rel_dataset
Rel122 [27]	Nouns	Relatedness	122	Rel122_dataset
SCWS [12]	Nouns	Relatedness	1994	SCWS1994_dataset
SimLex222 [11]	Verbs	Similarity	222	SimLex222_verbs_dataset
SimVerb3500 [8]	Verbs	Similarity	3500	Gerz_SimVerb3500_dataset
YP130 [29]	Verbs	Relatedness	130	Yang_YP130_dataset
WS353Full [7]	N, V, A	Relatedness	353	WordSim353Full_dataset
SimLex999 [11]	N,V,A	Similarity	999	SimLex999_dataset
MEN [4]	N,V,A	Relatedness	3000	MEN_dataset
RW2034 [16]	N,V,A	Relatedness	2034	RareWords2034_dataset
RW1401 [16]	N,V,A	Relatedness	2034	RareWords1401_dataset
SimLex111 [11]	Adjectives	Similarity	111	SimLex111_adjectives_dataset

Table 4 details all raw output data files of our experiments packaged into “*raw_output_benchmark_all_datasets.zip”* file which report the word similarity values obtained in the evaluation of all methods in all word similarity datasets. Finally, Table 5 details our processed output data files packaged into “*processed_output_benchmarks.zip”* file which contain the evaluation metrics as reported in data tables shown in our companion paper [1].

**Table 4:** Collection of raw output files generated by our reproducible experiment which are packaged into *raw_output_benchmark_all_datasets.zip* file as shown in Table 1. Each raw output file contains the raw similarity or relatedness values returned for each word pair in a specific word similarity or relatedness dataset (benchmark) by each semantic measure evaluated in our companion paper [1].

Dataset	Raw output filename (*.csv)
MC28 [19]	raw_similarity_values_MC28_dataset
RG65 [25]	raw_similarity_values_RG65_dataset
PS_full_[23]	raw_similarity_values_PSfull_dataset
Agirre201 [1]	raw_similarity_values_Agirre201_lowercase_dataset
SimLex665 [11]	raw_similarity_values_SimLex665_dataset
MTurk771 [10]	raw_similarity_values_MTurk771_dataset
MTurk287/235 [24]	raw_similarity_values_MTurk287-235_dataset
WS353Rel [7]	raw_similarity_values_WS353Rel_dataset
Rel122 [27]	raw_similarity_values_Rel122_dataset
SCWS [12]	raw_similarity_values_WS353Full_dataset
SimLex222 [11]	raw_similarity_values_SimLex111_dataset
SimVerb3500 [8]	raw_similarity_values_SimLex222_dataset
YP130 [29]	raw_similarity_values_SimLex999_dataset
WS353Full [7]	raw_similarity_values_SimVerb3500_dataset
SimLex999 [11]	raw_similarity_values_MEN_dataset
MEN [4]	raw_similarity_values_YP130_dataset
RW2034 [16]	raw_similarity_values_RareWords2034_dataset
RW1401 [16]	raw_similarity_values_RareWords1401_dataset
SimLex111 [11]	raw_similarity_values_SCWS1994_dataset

**Table 5:** Collection of processed output files packaged into “processed_output_benchmarks.zip” file which are generated by running the 'embeddings_vs_ontomeasures_final_tables.R' script file onto the output directory containing all raw data files shown in Table 4, together with their corresponding tables in our companion paper [1].

#	Post-processing output	In companion paper [1]
1	table_Pearson_SimDatasets.csv	Table 4 (full precision)
2	table_Pearson_SimDatasets_rounded.csv	Table 4
3	table_Spearman_SimDatasets.csv	Table 5 (full precision)
4	table_Spearman_SimDatasets_rounded.csv	Table 5
5	table_Pearson_RelDatasets.csv	table 6 (full precision)
6	table_Pearson_RelDatasets_rounded.csv	table 6
7	table_Spearman_RelDatasets.csv	table 7 (full precision)
8	table_Spearman_RelDatasets_rounded.csv	table 7
9	table_joined_allEmbeddings_similarity.csv	table 8 (full precision)
10	table_joined_allEmbeddings_similarity_rounded.csv	table 8
11	table_joined_allEmbeddings_relatedness.csv	table 9 (full precision)
12	table_joined_allEmbeddings_relatedness_rounded.csv	table 9
13	table_pvalues_AttractReppel_allembeddings_similarity.csv	table A.1 (appendix A)
14	table_pvalues_Paragramws_allembeddings_relatedness.csv	table A.2 (appendix A)
15	table_AvgMeasures_Pearson_SimDatasets.csv	table A.3 (full precision)
16	table_AvgMeasures_Pearson_SimDatasets_rouned.csv	table A.3 (appendix A)
17	table_AvgMeasures_Spearman_SimDatasets.csv	table A.4 (full precision)
18	table_AvgMeasures_Spearman_SimDatasets_rounded.csv	table A.4 (appendix A)
19	table_AvgMeasures_Pearson_RelDatasets.csv	table A.5 (full precision)
20	table_AvgMeasures_Pearson_RelDatasets_rounded.csv	table A.5 (appendix A)
21	table_AvgMeasures_Spearman_RelDatasets.csv	table A.6 (full precision)
22	table_AvgMeasures_Spearman_RelDatasets_rounded.csv	table A.6 (appendix A)

## 2 Experimental design, materials, and methods

Main aim of our aforementioned experiments was to carry-out the largest, unified and reproducible experimental study onto the state of the art in the families of ontology-based semantic similarity measures and Word Embedding (WE) models reported in the literature, together with a detailed and reproducible statistical significance analysis of the results. For this reason, we designed an experimental setup based on the software implementation of all methods evaluated in our experiments into a common software library called HESML [14]. HESML is a scalable and efficient self-contained Java software library of semantic measures based on WordNet whose latest version, called HESML V1R4 [13], also supports the evaluation of pre-trained word embedding files. HESML sets a self-contained experimentation platform on word similarity which is especially well suited to run large experimental surveys by supporting the running of automatic reproducible experiment files based on a XML-based file format, such as the ‘*benchmark_survey.exp*’ file detailed in Table 1.

**Fig. 1:**
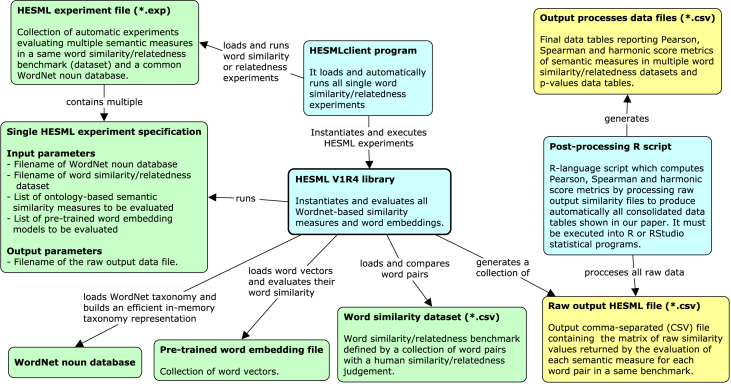
Concept map detailing our experimental setup to run automatically all experiments reported in our companion paper [14] and generate raw and processed data introduced herein. Input data files are shown in green, whilst output raw and processed data files are shown in yellow and software components are shown in blue. All reproducible experiments are specified into a single experiment file called ‘benchmark_survey.exp’ which is executed by HESMLclient program as detailed in section Appendix B.4.1. of ‘appendix-reproducible-experiments.pdf’ file. Both aforementioned files are detailed in Table 1.

All our experiments and raw output data were generated by running the HESMLclient program with the ‘*benchmark_reproducible.exp*’ file (see Table 1) as shown in Fig. 1. Likewise, the running of HESMLclient program was recorded with the Reprozip program [6] with the aim of generating the ‘*WN_ontology_measures_vs_embeddings.rpz*’ file detailed in Table 1. ReproZip is a virtualization tool whose aim is to warrant the exact replication of experimental results in the long-term by capturing and packaging into a same execution unit all experimentation program dependencies, being able to reproduce the packaged experiments onto any other platform regardless of the hardware and software configuration used in their creation. Thus, our aforementioned Reprozip file allows to reproduce our experiments in any platform supported by Reprounzip, which includes most Linux-based and Windows-based systems, regardless the software and hardware setup used in our experiments.

Fig. 1 shows a concept map detailing our experimental setup to run automatically all experiments and results reported in our companion paper [1]. Appendix B of our companion paper introduces a very detailed reproducibility protocol which explains how to use our reproducibility dataset [15] to reproduce all our experiments, as well as how to reproduce all output raw and processed data files detailed in Table 4, Table 5 from scratch. A copy of this later appendix is included in ‘*appendix-reproducible-experiments.pdf’* file detailed in Table 1.

## 3 Generation of our raw and processed data

Main raw output data provided by our dataset is a collection of files packaged into ‘raw_output_benchmark_all_datasets.zip‘ file which contain the raw similarity values obtained by the evaluation of all word similarity methods (see Table 1, Table 2 [1]) in all word similarity and relatedness benchmarks packaged into ‘*Word_Similarity_Dataset.zip*’ detailed in Table 3. Fig. 1 shows a concept map which allows to understand the experimental setup used to run our experiments and to generate all raw output similarity files as detailed in Table 4.

Main steps in the running of our experiments and generation of our raw output data are as follows:

(1) Experiments are generated by running the following HESMLclient.jar program with the ‘benchmark_survey.exp’ file as main input parameter, such as detailed in Appendix B.4.1 of our companion paper [1].
(2) HESMLclient program loads the *benchmark_reproducible.exp* experiment file to create an instance of a WordNet-based experiment object implemented by HESML library. Main input data to run any single experiment is shown in green in Fig. 1, and it is made up by the following information and input files:
  a. Filename of the WordNet [18] noun database used for the experiments. HESML library is distributed with three full versions of WordNet, versions 2.1, 3.0 and 3.1. Thus, any user could use any of them for his experiments; however, our experiments and data were generated with WordNet 3.0.
  b. Filename of the word similarity or relatedness dataset (benchmark) to be evaluated. These word similarity/relatedness benchmark are distributed with HESML, but they have been also gathered into the ‘Word_Similarity_Datasets.zip’ file, as detailed in Table 1, with the aim of simplifying their access to the research community by avoiding the download and installation of HESML library if it would not be needed.
  c. List of ontology-based semantic similarity measures based on WordNet.
  d. List of pre-trained word embedding models (files) to be evaluated. Because the large size of these later files, they are not distributed with HESML, neither in GitHub^1^ nor Mendeley repository [13]. Thus, we gathered all pre-trained models used in our experiments into the ‘*WordEmbeddings.zip*’ file (see Table 1) with the aim of warranting the permanent access to them, as well as the reproducibility of our data and experiments in the long term.
(3) WordNet-based HESML experiment object loads a WordNet database instance in memory and runs every single experiment by carrying-out the following tasks:
  a. To load in memory the word similarity dataset file containing the collection of word pairs whose semantic similarity or relatedness will be evaluated in the same experiment.
  b. To instance an object implementing every semantic measure specified for the single experiment.
  c. To evaluate and record the semantic similarity returned by each semantic measure for each word pair in the previously loaded word-similarity dataset.
  d. To build an in-memory matrix containing the word similarity returned by each semantic measure for each word pair.
  e. To write a raw output data file which contains the word similarity values for all word pairs included by each word similarity dataset as that shown in yellow in Fig. 1. Every single WordNet-based HESML experiment writes a single raw output similarity file in comma-separated (*.csv) file format for each word similarity dataset (benchmark) as detailed in Table 4.
(4) All raw data similarity files are loaded and processed by running the complementary R-script post-processing file (see Table 1) with the aim of computing all metrics reported in all data tables of our companion paper [1]. The running of our aforementioned R-script file into R or RStudio statistical packages generates all output processed files detailed in Table 5.

For a more detailed information on the use of our dataset and the replication of our experiments, we refer any reader to the appendix B of our companion paper [1].

## 4 Extending or modifying our experiments

Every word similarity or relatedness experiment specified in HESML platform is coded into a XML-based file, such as the ‘*benchmark_survey.exp*’ file detailed in previous section, and it is based on the definition of the collection of input parameters detailed in step 2 above. Thus, any user of our dataset could use it as a template to carry-out new experiments by editing this later experimentation file and selecting other ontology-based semantic measures currently implemented in HESML, as well as other word similarity datasets by providing new benchmarks in the same text-based CSV file format, or other unexplored pre-trained word embedding models by providing their vector files. For more detailed information, we refer the reader to the release notes of HESML V1R4 [13] and the original paper introducing HESML library [14]. Likewise, we invite any reader to subscribe to the HESML community forum for questions by sending an email to the hesml+subscribe@googlegroups.com address.

## Footnotes

1 nhttps://github.com/jjlastra/HESML.git.
